# Effect of Re-acidification on Buffalo Grass Rhizosphere and Bulk Microbial Communities During Phytostabilization of Metalliferous Mine Tailings

**DOI:** 10.3389/fmicb.2019.01209

**Published:** 2019-05-31

**Authors:** Linnea K. Hernandez, Catherine F. Gullo, Julia W. Neilson, Jon Chorover, Raina M. Maier

**Affiliations:** Department of Soil, Water, and Environmental Science, The University of Arizona, Tucson, AZ, United States

**Keywords:** phytostabilization, rhizosphere microbiota, re-acidification, plant growth-promoting bacteria, Fe/S-oxidizing bacteria, Fe-reducing bacteria, mine-tailing acidification

## Abstract

Phytostabilized highly acidic, pyritic mine tailings are susceptible to re-acidification over time despite initial addition of neutralizing amendments. Studies examining plant-associated microbial dynamics during re-acidification of phytostabilized regions are sparse. To address this, we characterized the rhizosphere and bulk bacterial communities of buffalo grass used in the phytostabilization of metalliferous, pyritic mine tailings undergoing re-acidification at the Iron King Mine and Humboldt Smelter Superfund Site in Dewey-Humboldt, AZ. Plant-associated substrates representing a broad pH range (2.35–7.76) were sampled to (1) compare the microbial diversity and community composition of rhizosphere and bulk compartments across a pH gradient, and (2) characterize how re-acidification affects the abundance and activity of the most abundant plant growth-promoting bacteria (PGPB; including N_2_-fixing) versus acid-generating bacteria (AGB; including Fe-cycling/S-oxidizing). Results indicated that a shift in microbial diversity and community composition occurred at around pH 4. At higher pH (>4) the species richness and community composition of the rhizosphere and bulk compartments were similar, and PGPB, such as *Pseudomonas*, *Arthrobacter*, *Devosia*, *Phyllobacterium*, *Sinorhizobium*, and *Hyphomicrobium*, were present and active in both compartments with minimal presence of AGB. In comparison, at lower pH (<4) the rhizosphere had a significantly higher number of species than the bulk (*p* < 0.05) and the compartments had significantly different community composition (unweighted UniFrac; PERMANOVA, *p* < 0.05). Whereas some PGPB persisted in the rhizosphere at lower pH, including *Arthrobacter* and *Devosia*, they were absent from the bulk. Meanwhile, AGB dominated in both compartments; the most abundant were the Fe-oxidizer *Leptospirillum* and Fe-reducers *Acidibacter* and *Acidiphilium*, and the most active was the Fe-reducer *Aciditerrimonas*. This predominance of AGB at lower pH, and even their minimal presence at higher pH, contributes to acidifying conditions and poses a significant threat to sustainable plant establishment. These findings have implications for phytostabilization field site management and suggest re-application of compost or an alternate buffering material may be required in regions susceptible to re-acidification to maintain a beneficial bacterial community conducive to long-term plant establishment.

## Introduction

Phytostabilization is a promising remediation strategy used to mitigate human-induced metal-contamination of the environment from legacy mine wastes through planting directly into the soil to achieve *in situ* contaminant-containment by reducing wind transport and promoting immobilization of metal(loid)s in the root zone (Gil-Loaiza et al., 2016, 2018). One particular area of concern is legacy mine sites that contain weathered and oxidized sulfide-rich tailings which are often contaminated with high levels of metal(loid)s remaining as a by-product of the mining process. The harsh conditions frequently associated with these mine tailings in addition to high metal(loid) content include acidic pH, low carbon and nutrient content, poor soil structure, and a lithoautotroph-dominated microbial community (Mendez et al., 2008; Valentín-Vargas et al., 2014), which together severely limit the potential for successful plant establishment for *in situ* metal(loid) containment. Research has demonstrated successful plant establishment in these materials following amendment with compost that provides a high proton consumption capacity (PCC), nutrients (i.e., carbon, nitrogen, phosphorous), and a heterotrophic microbial inoculum (Solís-Dominguez et al., 2011; Valentín-Vargas et al., 2014, 2018; Gil-Loaiza et al., 2016). The neutralizing effect of and presence of humic substances in organic amendment further serves to decrease bioavailability of metal(loid)s which can be toxic to pants (Wong and Selvam, 2006; Solís-Dominguez et al., 2011; Houben et al., 2013; Valentín-Vargas et al., 2014). Over time, however, a return to acidic conditions, or re-acidification, occurs if the acid-generating potential (AGP) of the substrate exceeds the PCC, posing an obstacle to long term-plant establishment.

The ideal goal of phytostabilization is the development of a sustainable “soil” characterized by a stable ecological state that is resistant to disturbances and capable of supporting long-term plant establishment (Santini and Banning, 2016). In the case of sulfidic mine tailings, this requires attainment of a circum-neutral pH with sufficient PCC to neutralize substrate AGP, and the development of a plant-supporting bacterial community with the capacity to limit biotic acid-generating activity. A typical sustainable soil representative of a stable ecological state is characterized by rhizosphere communities that represent an enrichment of species found in the bulk (Edwards et al., 2015; van der Heijden and Schlaeppi, 2015; Yeoh et al., 2017). Thus, the species diversity decreases from the bulk to the rhizosphere (Thirup et al., 2003; Buée et al., 2009; García-Salamanca et al., 2013; Edwards et al., 2015; Hacquard et al., 2015; Yeoh et al., 2017). Furthermore, this means that the bulk must contain a plant-supporting bacterial community as a resource from which the plant can recruit (Yeoh et al., 2017). Compost amendment provides this initial resource during mine-tailings phytostabilization. Factors that could disturb this balance and prevent the establishment of a stable ecological state include consumption of compost-introduced PCC due to high AGP, compost degradation, or compost-loss after weather events (i.e., rainfall and wind) prior to the establishment of a stable plant cover and heterotrophic microbial community.

Development of a sustainable “soil” requires an improved understanding of the microbial community dynamics associated with plant establishment and re-acidification processes in acidic, sulfidic mine-tailings and the application of this knowledge to successful phytostabilization management practices. Weathering of sulfides and consequential oxidation is a major driver of acidification. Furthermore, un-amended sulfidic mine tailings are dominated by iron (Fe)- and sulfur (S)-oxidizers (Mendez et al., 2007, 2008; Mendez and Maier, 2008; Schippers et al., 2010; Solís-Dominguez et al., 2011; Li et al., 2015), which play an increasingly larger role in acidification of the mine tailings as the pH decreases (Valentín-Vargas et al., 2014, 2018). Previous research has shown that organic amendments decrease the abundance and activity of resident acidophilic lithoautotrophic Fe/S oxidizers by (1) increasing the overall bacterial biomass (Gil-Loaiza et al., 2016) and diversity (Valentín-Vargas et al., 2018) and (2) altering the environment (i.e., increasing pH and organic carbon levels) to conditions less favorable to the pre-existing lithoautotrophic community (Johnson and Hallberg, 2008). In addition, the heterotrophic inoculum contains carbon-cycling bacteria vital to soil development, as well as plant-growth-promoting bacteria (PGPB) which (1) increase nutrient (i.e., phosphorous, iron, and nitrogen) availability; (2) decrease the plant-stress hormone ethylene through production of ethylene-cleaving ACC (1-aminocyclopropane-1-carboxylate) deaminase; (3) provide protection from pathogens; and (4) decrease metal bioavailability to plants (Bennisse et al., 2004; Tilak et al., 2005; Hayat et al., 2010). The establishment of a vegetative cover creates a self-sustaining positive feedback loop between the substrate material, the microbial community, and plants. Specifically, organic root exudates and plant litter nourish the developing PGPB community, maintain a buffered tailings substrate, augment soil organic matter, and keep the acid-generating lithoautotrophic community suppressed, all of which provide a favorable environment for plant growth.

Research on pyritic mine tailings has shown that establishment of a plant cover is associated with a shift in microbial populations as the pH reaches circum-neutral conditions (Moynahan et al., 2002; Mendez et al., 2007; Li et al., 2015, 2016), however, there are limited data on the bacterial-community dynamics associated with the re-acidification processes. In a long-term controlled greenhouse mesocosm study using pyritic metalliferous mine tailings from the Iron King Mine and Humboldt Smelter Superfund site (IKMHSS), we showed that pyrite weathering, the bioavailability of Fe and As, and microbial community diversity and phylogenetic composition are closely associated with pH (Valentín-Vargas et al., 2014, 2018). In the absence of plants, tailings acidification was associated with a temporal progression of Fe- and S-oxidizing bacteria and archaea beginning with bacterial species sensitive to low-pH and progressing to communities dominated by acid-tolerant *Leptospirillum* and *Ferroplasma* species (Valentín-Vargas et al., 2018). While plant establishment impacted the microbial community composition and imposed a control on the acidification process, pH was identified as the most significant environmental variable influencing the community composition of the rhizosphere bacterial, archaeal, and fungal populations. Further, decreasing pH was associated with increasing pore water concentrations of labile metal(loids), and Co, Cd, Pb, and As were found to have significant impacts on the community composition of bacteria, archaea, and fungi (Valentín-Vargas et al., 2014). Thus, pH served as a comprehensive indicator of both direct and indirect (i.e., metal toxicity) impacts on microbial community structure during tailings acidification.

In a parallel IKMHSS field study, associations between pH and the composition of bacterial communities colonizing the root surface (rhizoplane) of buffalo grass during different stages of compost-assisted phytostabilization were documented (Honeker et al., 2017). *Alphaproteobacteria* relative abundance was negatively associated with pH in a compost-eroded area that had undergone extensive re-acidification, however, was positively associated with pH in a less disturbed area that maintained a circum-neutral pH. This difference was also reflected in the composition of the associated rhizosphere communities; *Alphaprotebacteria* in the rhizosphere were dominated by the heterotrophic, acidophilic genus *Acidiphilium* in the area effected by re-acidification, and by *Rhizobiaceae*, a family containing many neutrophilic PGPB, in the circum-neutral region. Taken together, the results from previous field and greenhouse studies demonstrate the strong association between pH and microbial community composition during the weathering of un-vegetated tailings. Similarly, pH served as a strong indicator of microbial community dynamics on the root surface and in the rhizosphere of plants during phytostabilization. We contend that pH in this system is the best overall indicator of re-acidification and the associated changes in microbial communities that drive the weathering of Fe- and S-minerals.

The current study builds on this previous work by evaluating the effect of re-acidification status, as measured by substrate pH, on the diversity, composition, and activity of microbial communities in bulk tailings and rhizosphere during phytostabilization with buffalo grass at the field-scale. Specifically, the objectives are to (1) compare microbial communities and diversity between rhizosphere and bulk compartments and (2) characterize how re-acidification affects the abundance and activity of the most prominent PGPB (including N_2_-fixing) and Fe-cycling/S-oxidizing (acid-generating) bacteria (AGB). The results of this study describe how re-acidification affects the dynamics and recruitment of plant-associated microbial communities, which has implications for the management of phytostabilization sites.

## Materials and Methods

### IKMHSS Field Site and Study Description

The Iron King Mine and Humboldt Smelter Superfund (IKMHSS) site is located adjacent to the town of Dewey-Humboldt, AZ. Mining operations were carried out between the late 1800s and 1969 extracting Cu, Ag, Au, Pb, and Zn (Creasey, 1952). A 64 ha residual tailings pile was left behind after mining closure containing high concentrations of toxic metal(loid)s including As, Pb, and Zn at 3.1, 2.2, and 2.6 g kg^-1^ tailings, respectively (Root et al., 2015). A complete mineralogical characterization of the IKMHSS tailings was previously reported (Hayes et al., 2014; Root et al., 2015) and revealed an extremely acidic (pH 2.3–3.7) oxidized surface layer (0–25 cm depth) rich in sulfate minerals (e.g., jarosite, gypsum) and iron-oxides (e.g., ferrihydrite), overlying neutral (pH 6.3–7.3), unoxidized subsurface material (>35 cm depth) rich in iron sulfides (e.g., pyrite). In the oxidized top layer (0–25 cm) the elements with highest bioavailability (aqueous extractable) are Mg (0.9 g kg^-1^), Al (1.7 g kg^-1^), Ca (17.2 g kg^-1^), and Fe (2.4 g kg^-1^) (Hayes et al., 2014). Additionally, the tailings are characterized by low organic carbon content (0.14 g kg^-1^), hypersalinity (EC 6.5–9.0 ds m^-1^), and poor substrate structure (Hayes et al., 2014; Valentín-Vargas et al., 2014) contributing to the complete absence of vegetation.

The IKMHSS field trial consists of three phases implemented in consecutive years to evaluate multiple direct planting strategies for native plant species in compost-amended tailings. Phase 1, described in detail in Gil-Loaiza et al. (2016), was initiated in May 2010 to study different compost amendment rates (10, 15, and 20% [w/w in the top 20 cm]) and was seeded with six native plant species in semi-random plots. For the current study, rhizosphere and bulk samples associated with buffalo grass grown in the 15% compost treatment were examined (Plots 5, 10, 19, and 24). Phase 3, initiated in June 2012, was designed to study the effectiveness of buffalo grass or quailbush monocultures situated in rows. The entire area was amended with 15% compost (w/w in the top 20 cm) and lime (2.1 kg m^-2^). Annual sampling indicated that the previously characterized Fe/S mineral assemblage and toxic metal(loid) speciation profiles (Hayes et al., 2014; Root et al., 2015) are representative of both Phases 1 and 3 substrates (Robert Root; personal communication). For this study, rhizosphere and bulk samples associated with buffalo grass plants from the three replicates of the buffalo grass treatment were examined (Rows 2, 4, and 6). For Phase 1, the AGP of the tailings was 35.1 kg CaCO_3_ ton^-1^ at time of implementation (2010) and 23.4 kg CaCO_3_ ton^-1^ in the year of sample collection (2013), equivalent to pyrite levels of 2.1 and 1.4%, respectively (*data not published*). The presence of pyrite and its associated AGP indicate that 15% compost amended tailings had the potential to produce acid. AGP was not calculated for Phase 3.

### Sample Collection and Processing

Rhizosphere and bulk samples were collected from eight buffalo grass plants from Phase 1 (Plot 5a and b, Plot 10a and b, Plot 19a and b, and Plot 24a and b) and eight from Phase 3 (Row 2a and b; Row 4a, b, and c; and Row 6a, b, and c) in 2013. Briefly, the top of the plant was cut off at the surface of the substrate, and a corer measuring 10 cm in length and 2 cm in diameter was inserted directly over the truncated root and the substrate core was placed into a sterile bag for rhizosphere DNA and RNA analysis. An additional substrate core was collected 10–15 cm from the plant, in a direction with no plant cover, for bulk DNA and RNA analysis. For each sampling location, we also measured plant chlorophyll and cover as described previously (Honeker et al., 2017). For substrate geochemical analysis, a surface tailings sample from the top 20 cm was collected for each plant between the rhizosphere and bulk sampling locations. Samples were immediately stored on ice for transport to the lab (5–15 h). Immediately upon arrival to the lab, 0.5 and 0.25 g subsamples from the cores were placed into lysis tubes for DNA and RNA extractions, respectively. For RNA preservation, 0.5 mL of Life Guard Soil Preservation Solution (MO BIO Laboratories, Inc., Carlsbad, CA, United States) was added to the RNA extraction lysis tubes. One subsample for Row 2b.R (rhizosphere) for RNA extraction was lost. Lysis tubes with substrate material were stored at -80°C until DNA and RNA extraction.

### Geochemical Analysis

Rhizosphere tailings were analyzed for pH, total organic carbon (TOC), total nitrogen (TN), and electrical conductivity (EC). Each rhizosphere sample was sieved at 2 mm and dried at 65°C for 72 h. The pH and EC were measured on the aqueous phase of a 1:2 mass ratio paste of tailings to ultrapure DI water (18.2 MΩ, Milli-Q) that was mixed for 30 min prior to measurement. The pH was measured in the homogenized paste and the EC was determined from the supernatant after the substrate was allowed to settle for 10–15 min. Analyses of TOC and TN were performed on milled subsamples of the dried rhizosphere material (Shimadzu TOC analyzer, Columbia, MD, United States) using a solid state module (SSM), which utilizes a dry combustion under oxygen with detection by a non-dispersive infrared gas analyzer (NDIR) for total carbon and chemoluminescence for TN.

### Extraction of Nucleic Acids

DNA extractions were performed using the FastDNA Spin Kit for Soil (MP Biomedicals; Santa Ana, CA, United States) following the manufacturer’s protocol with modifications to enhance DNA yield, as outlined in Valentín-Vargas et al. (2014). Substrate samples were thawed on ice prior to extraction, and all tubes, pipettes, and pipette tips were UV sterilized for 30 min. DNA concentrations were measured using the Qubit 3.0 Fluorometer (Thermo Fischer Scientific, Waltham, MA, United States). To maximize success of downstream sequencing, we found a combination of clean-up using the ZR Genomic Clean and Concentrator-10 kit (ZymoResearch Corporation, Irvine, CA, United States) and dilution, if needed, to a final concentration of less than 10 ng/μL to be optimal.

RNA extractions were performed using the ZR Soil/Fecal RNA MicroPrep kit (ZymoResearch Corporation, Irvine, CA, United States) following modifications outlined in Nelson et al. (2015). Prior to RNA extraction, all surfaces and pipettes were pre-treated with RNaseZap Wipes (Ambion, Grand Island, NY, United States). Substrate samples were thawed on ice prior to extraction and centrifuged to remove the LifeGuard Soil Preservation solution. Residual DNA was removed with a 35-min DNase treatment at 37°C, as described in Neilson et al. (2010). RNA quality was checked with gel electrophoresis and quantified using the TBS-380 Fluorometer (Turner BioSystems, Sunnyvale, CA, United States) in conjunction with the Quant-it RNA RiboGreen quantification kit (Invitrogen, Carlsbad, CA, United States) following the manufacturers’ protocols. RNA samples for Plot 24b.R, Plot 24b.B (bulk), Row 2c.R, and Row 2c.B had undetectable RNA concentrations and thus could not be analyzed.

### Reverse Transcriptase PCR (RT-PCR)

First strand cDNA synthesis was performed on extracted RNA using the SuperScript III First Strand Synthesis System for RT-PCR (Invitrogen-Thermo Fischer Scientific, Waltham, MA, United States), which uses random hexamer primers, following the manufacturer’s protocol. Second strand synthesis was performed using the NEB mRNA Second Strand Synthesis Module (New England BioLabs, Ipswich, MA, United States), following the manufacturer’s protocol. Double-stranded cDNA was cleaned up using the Qiagen Mini Elute PCR Purification Kit (Qiagen, Hamburg, Germany), following the manufacturer’s protocol. Final double-stranded cDNA was quantified using the Qubit 3.0 Fluorometer (Thermo Fischer Scientific, Waltham, MA, United States). Due to overall low concentrations of double-stranded cDNA (<10 ng/μL), no dilutions were required.

### 16S rRNA Gene Amplicon Sequencing

Final DNA and double-stranded cDNA samples were sent to Argonne National Laboratory (Argonne, IL, United States) for library preparation and amplicon sequencing. Library preparation followed a single-step PCR protocol presented by Caporaso et al. (2012) using primers 505f and 806r to amplify the V4 region of the 16S rRNA gene. Sequencing was carried out in a single lane on an Illumina MiSeq using v2 chemistry and bidirectional amplicon sequencing (2 × 151 bp).

Raw sequence reads were processed using the open source software package QIIME v1.9 (Quantitative Insights into Microbial Ecology^1^). Forward and reverse reads were joined using a minimum overlap of 30 bp, followed by quality filtering using default QIIME parameters, and demultiplexing. Default QIIME quality filtering parameters include: removal of the primer/barcode from the sequence, and removal of all sequences (1) with a Phred quality score <4, (2) containing any ambiguous (N) base calls, and (3) having a length of < 0.75 of original length post-quality filtering. After quality filtering, a total of 7,150,517 sequence reads remained, with an average of 121,195 ± 27,274 sequence reads per sample and a median sequence length of 253 bp. Using a sequence cutoff of 30,000 reads, one sample (Row 4b.R RNA) was removed from further analysis. Sequences were clustered into 553,627 operational taxonomic units (OTUs) at 97% or greater sequence similarity using UCLUST (Edgar, 2010). Representative sequences from each OTU were aligned using PyNAST (Caporaso et al., 2010) and assigned taxonomy using the UCLUST classifier and Greengenes 16S rRNA gene database (DeSantis et al., 2006). During DNA extraction, blanks that contained no tailings sample were extracted alongside each batch of samples for quality control and subsequently sequenced. For DNA samples, OTUs contained in the sequenced blanks were removed from the associated samples extracted in the same batch to minimize influence of contamination. RNA sample blanks contained a high number of OTUs which were also present in the samples, thus, it was concluded that the contamination was acquired from nearby samples during either the RNA extraction or cDNA library construction steps. To address this, each OTU was individually assessed, and if it was not present in the associated DNA samples (post-contaminant screening), it was removed from all the samples extracted in the same batch. Low abundant (less than 0.001% of total) OTUs were removed and OTU tables were rarefied to 50,000 sequence reads, prior to downstream analyses.

### rRNA:rRNA Gene Ratios

The 16S rRNA and 16S rRNA gene datasets were used to calculate rRNA:rRNA gene ratios for specific OTUs that represented the most abundant (out of the top 100 OTUs) putative PGPB (including neutrophilic N_2_-fixers) and Fe/S-cyclers and S-reducers (Fe-reducers, Fe-oxidizers, Fe/S-oxidizers, and S-oxidizers), collectively representing a microbial community conducive to plant growth vs. an acid-generating community not capable of supporting plant growth. Putative function was assessed by BLAST (National Institute of Health^2^) searches of the OTU sequences to determine the taxonomy of the closest match (>93% for genus and >96% for species identity) and a subsequent literature search for functional capabilities. Assignment of functional capacity was based on experimental verification of the function and/or the presence of the gene capable of the function, as published in the literature.

rRNA:rRNA gene ratios have been used as a proxy for activity or growth rate in prior studies (Kerkhof and Ward, 1993; Muttray and Mohn, 1999; Ka et al., 2001; Pérez-Osorio et al., 2010; Campbell et al., 2011; Gaidos et al., 2011; Brettar et al., 2012; Campbell and Kirchman, 2013; Hunt et al., 2013; Gómez-Silván et al., 2014; Mei et al., 2016; Mueller et al., 2016) with the assumption that the rRNA in a cell is proportional to activity and that the number of rRNA genes are consistent across taxa being compared. Because these assumptions are more accurately applied to a single taxon, this study assessed activity through quantification of rRNA:rRNA gene ratios for a single OTU across different samples. rRNA:rRNA gene ratios were calculated by dividing the rarefied number of rRNA sequence reads of each OTU of the RNA by that of the DNA dataset.

### nifH Gene qPCR

Bacterial *nif*H gene was quantified to assess the nitrogen-fixing potential of the bacterial communities. The bacterial *nif*H gene, encoding the Fe-subunit of the nitrogenase enzyme, was amplified using primers PolF/PolR (Poly et al., 2001). These primers were designed to target diazotrophic soil bacteria (Poly et al., 2001) and have been shown to successfully target *nifH* from a diverse range of diazotrophic bacteria (Wartiainen et al., 2008). Furthermore, this primer pair has been shown to be effective in conjunction with qPCR (Nelson et al., 2015; Bouffaud et al., 2016). Representative sequences for qPCR calibration curves were previously determined using clone libraries produced from the IKMHSS substrate, by selecting clones from the most abundant taxonomic group for each gene and preserving the plasmids (Nelson et al., 2015). Calibration curves were prepared by 10-fold serial dilutions of plasmids. Triplicate 10 μL reactions were amplified using a CFX96 Real-Time Detection System (Bio-Rad Laboratories, Hercules, CA, United States) containing: 1 μL template, 5 μL SsoFast EvaGreen Supermix (Bio-Rad Laboratories, Hercules, CA, United States), and 0.4 μM of each primer. Reaction conditions were as follows: 95°C for 1 min followed by 50 cycles of 95°C for 6 s and 59°C for 6 s qPCR efficiency was ≥85% (r_2_ = 0.99). Following amplification, a melt curve was created under the following conditions: 95°C for 10 s, 5 s cycles increasing from 65 to 90°C in 0.5°C increments, and a final extension at 72°C for 5 min. The melt curve was performed to confirm product specificity and aided in determination of the detection limit in conjunction with the standard curve. Minimum and maximum detection thresholds were set based on the cycle numbers (Cq) of the highest and lowest reproducible standard curve, the Cq of the DNA extraction blanks processed with respective samples, the Cq of the qPCR negative controls, and deviations from a melt curve representing *nif*H amplification. Samples below the detection limit were assigned a Cq value equal to the average of the Cq for the DNA extraction blanks to account for extraction error.

### Statistical Analysis

For correlation analysis between microbial communities and geochemical conditions, Spearman correlation coefficients (ρ) and *p*-values were calculated using the program PAST (Hammer et al., 2001) with a subsequent false discovery rate (FDR) analysis with an α of 0.05. A linear discriminant analysis (LDA) Effect Size (LEfSe) (Segata et al., 2011)^3^ method was performed to determine significant enrichments in rhizosphere versus bulk microbial communities at high pH (>4) and low pH (<4). Beta diversity was evaluated using principal coordinates analysis (PCoA) based on the unweighted UniFrac distance matrices, and analysis was done in QIIME. Significant differences between high and low pH bulk and rhizosphere samples were determined using a one-way ANOVA followed by a Tukey test for means comparisons, using the program OriginPro 2016 for alpha diversity, and PERMANOVA using QIIME for beta diversity.

## Results

### Geochemical

The pH, TOC, TN, and EC data associated with each buffalo grass plant are reported in Table 1. Recall that Phase 1 represents 3 years of plant growth and Phase 3 represents 1 year of plant growth. For reference, Phase 1 at time zero and after 1 year of growth (2011) had an average pH of 6.5 ± 1.1 (range: 5.08–7.75) and 7.0 ± 0.72 (range: 5.93–7.52), respectively (see 15% compost and seeds treatment in Gil-Loaiza et al., 2016). In comparison, Phase 3 at time zero and after 1 year of growth had an average pH of 7.4 ± 0.27 (range: 6.96–7.62) (*unpublished data*) and 6.2 ± 1.9 (range: 2.56–7.76) (Table 1), respectively. After 3 years of growth, Phase 1 had an average pH of 3.7 ± 1.3 (Table 1). This illustrates three important patterns in pH: (1) Phase 3 after 1 year of growth had a comparable pH to Phase 1 after 1 year of growth; (2) Phase 3 began to undergo some re-acidification after 1 year of growth due to flooding and compost erosion; and (3) Phase 1 had undergone extensive re-acidification after 3 years of growth. The data reveals the presence of a pH gradient for both Phases 1 and 3, and confirms that re-acidification of the planted mine tailings occurred over time in both Phase 1 and Phase 3. For many of the analyses in this paper, the samples are divided into two significantly distinct groups (*t*-test; *p* < 0.001): high pH (>4) and low pH (<4). Samples that fall into the high pH category are Plot 10a, Plot 10b, Row 2a, Row 2c, Row 4a, Row 4c, Row 6a, and Row 6b. Samples that fall into the low pH category are Plot 5a, Plot 5b, Plot 19a, Plot 19b, Plot 24a, Plot 24b, and Row 2b.

**Table 1:** Geochemical and plant condition parameters for tailings substrate collected next to each buffalo grass plant.

Sample	Phase	pH	TOC (g kg^-1^)	TN (g kg^-1^)	EC (dS m^-1^)	Chlorophyll	Plant cover (%)
Plot 24b	1	2.35	47.6	3.60	10.20	10.5	3.5
Plot 19b	1	2.47	51.1	3.46	4.46	11.4	49.3
Plot 5a	1	2.88	35.2	3.07	3.42	35.5	46.6
Plot 19a	1	3.46	44.6	3.66	2.97	38.6	49.3
Plot 24a	1	3.49	57.1	3.76	2.93	49.3	3.5
Plot 5b	1	3.66	39.6	3.29	3.17	12.8	46.6
Plot 10b	1	5.38	49.4	4.50	5.47	15.4	37.9
Plot 10a	1	6.01	72.2	5.22	7.94	32.3	37.9
Row 2b	3	2.56	47.7	4.52	14.56	7.5	29.4
Row 4b	3	4.71	48.7	5.78	5.76	11.6	29.5
Row 2c	3	5.22	51.9	5.62	4.30	8.7	29.4
Row 6a	3	6.54	99.2	9.04	8.10	50.0	73.2
Row 6b	3	7.11	68.4	7.13	9.46	8.3	29.5
Row 4c	3	7.72	126.8	9.54	9.83	31.1	65.7
Row 2a	3	7.73	153.6	9.53	4.10	40.5	48.4
Row 4a	3	7.76	140.8	8.68	8.86	44.2	53.6


*TOC, total organic carbon; TN, total nitrogen; EC, electrical conductivity.*

A range of values was also observed for the geochemical parameters TOC, TN, and EC (Table 1). TOC and TN were significantly greater in the high pH samples than the low pH samples (*t*-test; *p* < 0.05 and *p* < 0.001, respectively). Furthermore, pH was significantly correlated with TOC (*r* = 0.83, *p* < 0.001) and TN (*r* = 0.9, *p* < 0.0001) reflecting a strong association between these parameters. Therefore, the remainder of this paper will focus on pH to evaluate the specific effects of acidification.

### Microbial Communities

#### Diversity

**FIGURE 1:**
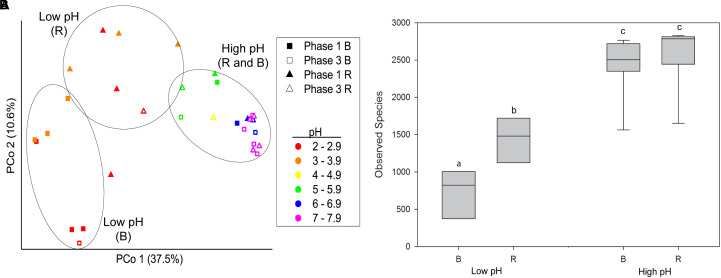
**(A)** Beta diversity of bacterial communities from buffalo grass bulk and rhizosphere substrate collected from Phase 1 and Phase 3 represented as a PCoA plot of unweighted UniFrac distances between all samples. Samples from rhizosphere at low pH (<4), bulk at low pH, and rhizosphere and bulk at high pH (>4) form three distinct groups (*p* < 0.05, PERMANOVA), as indicated by black circles. **(B)** Alpha diversity of microbial communities from buffalo grass bulk and rhizosphere samples from high pH (>4) and low pH (<4) substrate. Different letters indicate significant difference (ANOVA, Tukey’s HSD, *p* < 0.05). B, bulk; R, rhizosphere.

Beta diversity analysis of rhizosphere and bulk samples from Phase 1 and Phase 3 confirmed that pH was a major explanatory variable associated with the phylogenetic composition of microbial communities and that more variability existed between rhizosphere and bulk samples at low compared to high pH (Figure 1A). PCoA of beta diversity measured as unweighted UniFrac distances revealed that pH was a major source of variation across the x-axis, which explained 37.5% of variation. Three distinct groups were observed: (1) Bulk at low pH, (2) rhizosphere at low pH, and (3) rhizosphere and bulk at high pH (*p* < 0.05, PERMANOVA).

Thus, a significant rhizosphere effect on community composition was evident at low pH that was not observed at high pH. This pattern was also evident for the alpha diversity; at high pH, the alpha diversity, as measured by the number of observed species, was the same for rhizosphere and bulk samples (ANOVA and Tukey’s HSD, *p* > 0.05) (Figure 1B), whereas at low pH the alpha diversity was significantly higher in the rhizosphere compared to the bulk (ANOVA and Tukey’s HSD, *p* < 0.05). The alpha diversity in both the rhizosphere and bulk at high pH was significantly higher than the rhizosphere and bulk at low pH (ANOVA and Tukey’s HSD, *p* < 0.05).

#### Phylogenetic Profiles

**FIGURE 2:**
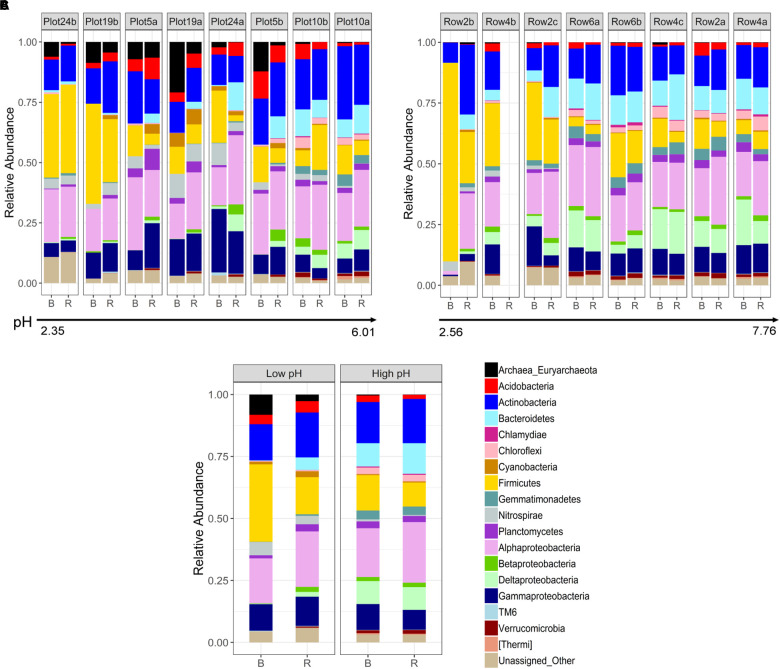
Phylogenetic profiles of **(A)** Phase 1 and **(B)** Phase 3 rhizosphere and bulk samples collected from buffalo grass plants used to phytostabilize pyritic metalliferous mine tailings, in order of increasing pH. For explanation regarding missing data (Row4b.R), please see Methods section. **(C)** Averaged phylogenetic profiles across low pH (<4) and high pH (>4) rhizosphere and bulk samples. B, bulk; R, rhizosphere.

Differences in phylogenetic community composition between rhizosphere and bulk compartments were evaluated for Phase 1 and 3 samples. The phylogenetic profiles are shown in Figure 2A,B, both ordered in ascending pH. In the rhizosphere and bulk, a strong and significant positive correlation (Spearman coefficient) was observed between pH and *Bacteroidetes*, *Chlamydia*, *Chloroflexi*, *Gemmatimondetes*, *Deltaproteobacteria*, *Verrucomicrobia*, and [*Thermi*], and a significant negative correlation with *Euryarchaeota* and *Nitrospirae* (Supplementary Figure 1). Additionally, in the bulk, pH was significantly positively correlated with *Cyanobacteria*, *Planctomycetes*, *Betaproteobacteria*, and *TM6*, and significantly negatively correlated with *Firmicutes* (Supplementary Figure 1). *Alphaproteobacteria* did not show any significant changes across the pH spectrum, however, at the family level there was a large shift from a dominance of *Acetobacteraceae* (primarily *Acidiphilium*) at low pH to a more diverse community dominated by *Rhizobiales* including *Hyphomicrobiaceae, Phyllobacteraceae, Bradyrhizobiaceae*, and *Rhizobiaceae*. Correlations between individual phyla and TOC where similar to those observed for pH, with the addition of a significantly negative correlation with *Cyanobacteria* (Supplementary Figure 1).

**FIGURE 3:**
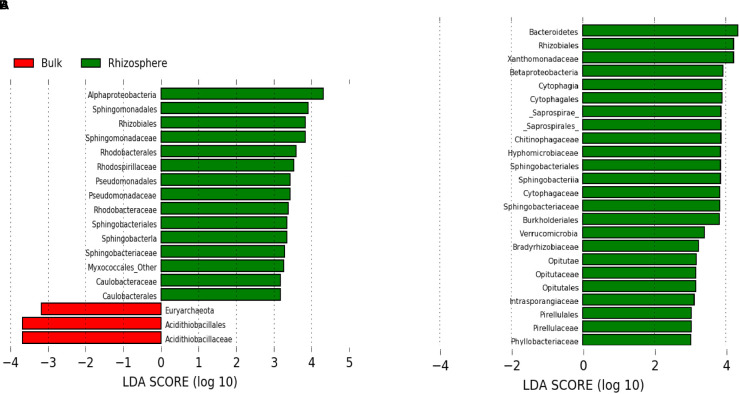
Microbial groups that show enrichment in the rhizosphere (green) or in the bulk (red) based on LEfSe results at **(A)** high pH (>4) (*p* < 0.05) and **(B)** low pH (<4) (*p* < 0.005). LDA threshold was set to 3.0.

As suggested by the beta diversity analysis, differences in rhizosphere and bulk microbial communities were more pronounced at low compared to high pH (Figure 2C). At high pH, communities did not differ significantly at the phylum/subphylum level, whereas at low pH, *Bacteroidetes*, *Verrucomicrobia*, and [*Thermi*] were significantly more abundant in the rhizosphere compared to the bulk (*p* < 0.05). This same pattern is supported by LEfSe analysis which revealed fewer phylogenetic groups at the order and family level enriched in the rhizosphere compared to bulk for high pH (Figure 3A) than for low pH substrates (Figure 3B). At high pH, top groups that showed enrichment in the rhizosphere (*p* < 0.05) included several groups within the subphylum *Alphaproteobacteria*, including *Sphingomonadales*, *Rhodobacterales*, *Rhizobiales*, and *Caulobacterales* at the order level and *Sphingomonadaceae, Rhodospirillaceae, Rhodobacteraceae*, and *Caulobacteraceae* at the family level. Other groups that were enriched in the rhizosphere were *Sphingobacteria*, *Pseudomonadales*, and *Myxococcales* (Figure 3A). A couple of groups showed enrichment in the bulk at high pH, including the Fe-oxidizing *Acidithiobacillaceae* of the order *Acidithiobacillales* and *Euryarchaeota* (Figure 3A). At low pH, over 100 phylogenetic groups were significantly discriminative between the rhizosphere and bulk, more than could be handled by the program. Again, this result highlights the higher variability between rhizosphere and bulk samples at low pH. To reduce the number of significantly discriminative microbial groups, the LEfSe analysis was repeated for the low pH substrates using a more stringent *p*-value cutoff of 0.005. At low pH, phylogenetic groups that showed enrichment in the rhizosphere that were also enriched at high pH were *Rhizobiales* and *Sphingobacteriales*. Specific *Rhizobiales* families that were enriched in the rhizosphere at low pH that were not enriched at high pH were *Hyphomicrobiaceae*, *Phyllobacteraceae*, and *Bradyrhizobiaceae*. Other groups enriched in the rhizosphere at low pH were *Saprospirae, Cytophagia, Intrasporangiaceae, Opitutatae, Xanthomonadaceae, Burkholderiales*, and *Pirellulales* (Figure 3B). At low pH, there were no microbial groups that where enriched in the bulk. Despite using the more stringent *p*-value cutoff for the low pH rhizosphere and bulk samples, there were still more groups that were enriched in the rhizosphere compared to the high pH.

### Fe-Cyclers/S-Oxidizers

#### Abundance vs. Activity

**FIGURE 4:**
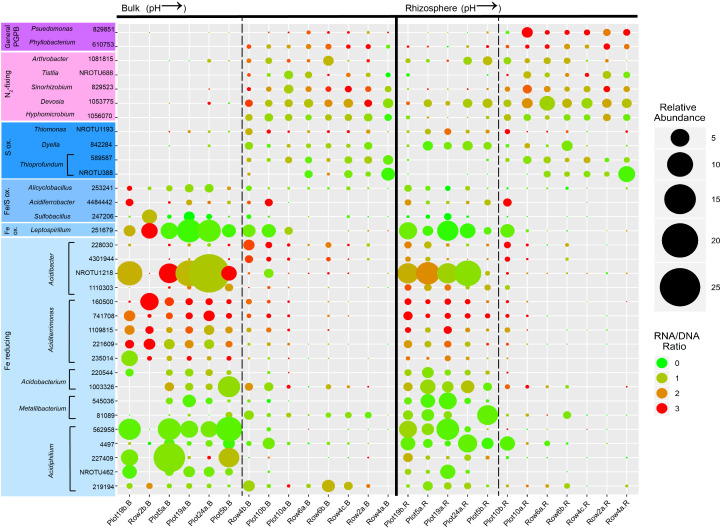
Bubble plot showing the relative abundance (depicted by size) and activity (depicted by color) of the most abundant OTUs present in the bulk and rhizosphere of buffalo grass that are putative plant-growth promoting, N_2_-fixing, S-oxidizing, Fe-oxidizing, and Fe-reducing bacteria. Putative functions are based on the known function of the closest BLAST database match for each OTU. Samples are ordered in ascending pH of bulk (on the left) and rhizosphere (on the right). The dashed lines represent pH 4. PGPB, plant growth-promoting bacteria.

The rRNA:rRNA gene ratio was calculated for phylotypes of interest as a proxy for the relative activity of different members of the community. OTUs representing putative Fe-cyclers/S-oxidizers, as predicted based on known functional capacities of closest BLAST matches, were selected from the top 100 most abundant OTUs (Table 2). The abundance and relative activity of these OTUs are displayed in Figure 4. It is evident that the most abundant OTUs were not always the most active members of the community at the time of sampling.

**Table 2:** Identities of putative N_2_-fixing, plant growth-promoting bacteria (PGPB), Fe/S oxidizing, and Fe reducing within the top 100 most abundant OTUs.

OTU	Closest BLAST match (accession no.)	%	Phylum	Putative function	References
829851	*Pseudomonas putida* (NR_11365.1)	99	*Gammaproteobacteria*	PGPB	Patten and Glick, 2002; Glick, 2004, 2014
1081815	*Arthrobacter pascens* (NR_026191.1)	100	*Actinobacteria*	PGPB	Grandlic et al., 2008; Langella et al., 2014
610753	*Phyllobacterium loti* (NR_133818.1)	98	*Alphaproteobacteria*	PGPB	Flores-Felix et al., 2015
NROTU688	*Tistlia consotensis* (NR_116437.1)	98	*Alphaproteobacteria*	N_2_ Fix.	Díaz-Cárdenas et al., 2010
829523	*Sinorhizobium medicae* (NR_104719.1)	99	*Alphaproteobacteria*	N_2_ Fix.	Rome et al., 1996; Galleguillos et al., 2000
1053775	*Devosia neptuniae* (NR_028838.1)	97	*Alphaproteobacteria*	N_2_ Fix.	Rivas et al., 2002
1056070	*Hyphomicrobium aestuarii* (NR_104954.1)	99	*Alphaproteobacteria*	N_2_ Fix.	Fan et al., 2018
NROTU1193	*Thiomonas arsenivorans* (NR_115341.1)	96	*Betaproteobacteria*	S/As Ox.	Battaglia-Brunet et al., 2011
842284	*Dyella thiooxydans* (NR_116006.1)	99	*Gammaproteobacteria*	S Ox.	Anandham et al., 2008, 2014
589587	*Thioprofundum hispidum* (NR_112620.1)	98	*Gammaproteobacteria*	S Ox.	Mori et al., 2011
NROTU388	*Thioprofundum hispidum* (NR_112620.1)	96	*Gammaproteobacteria*	S Ox.	Mori et al., 2011
253241	*Alicyclobacillus cycloheptanicus* (NR_118875.1)	98	*Firmicutes*	Fe/S Ox.	Johnson et al., 2003
4484442	*Acidiferrobacter thiooxydans* (NR_114629.1)	97	*Gammaproteobacteria*	Fe/S Ox.	Hallberg et al., 2011
247206	*Sulfobacillus acidophilus* (NR_074758.1)	96	*Fimicutes*	Fe/S Ox.	Baker and Banfield, 2003; Chen et al., 2016
251679	*Leptospirillum ferriphilum* (NR_028818.1)	99	*Nitrospirae*	Fe Ox.	Baker and Banfield, 2003; Mendez et al., 2007; Chen et al., 2016
228030	*Acidibacter ferrireducens* (NR_126260.1)	98	*Gammaproteobacteria*	Fe Red.	Falagán and Johnson, 2014
4301944	*Acidibacter ferrireducens* (NR_126260.1)	98	*Gammaproteobacteria*	Fe Red.	Falagán and Johnson, 2014
NROTU1218	*Acidibacter ferrireducens* (NR_126260.1)	98	*Gammaproteobacteria*	Fe Red.	Falagán and Johnson, 2014
1110303	*Acidibacter ferrireducens* (NR_126260.1)	100	*Gammaproteobacteria*	Fe Red.	Falagán and Johnson, 2014
160500	*Aciditerrimonas ferrireducens* (NR_112972.1)	93	*Actinobacteria*	Fe Red.	Itoh et al., 2011
741708	*Aciditerrimonas ferrireducens* (NR_112972.1)	93	*Actinobacteria*	Fe Red.	Itoh et al., 2011
1109815	*Aciditerrimonas ferrireducens* (NR_112972.1)	94	*Actinobacteria*	Fe Red.	Itoh et al., 2011
221609	*Aciditerrimonas ferrireducens* (NR_112972.1)	94	*Actinobacteria*	Fe Red.	Itoh et al., 2011
235014	*Aciditerrimonas ferrireducens* (NR_112972.1)	96	*Actinobacteria*	Fe Red.	Itoh et al., 2011
220544	*Acidobacterium capsulatum* (NR_074106.1)	96	*Acidobacteria*	Fe Red.	Kishimoto et al., 1991
1003326	*Acidobacterium capsulatum* (NR_074106.1)	97	*Acidobacteria*	Fe Red.	Kishimoto et al., 1991
545036	*Metallibacterium scheffleri* (NR_118103.1)	100	*Gammaproteobacteria*	Fe Red.	Ziegler et al., 2013
81089	*Metallibacterium scheffleri* (NR_118103.1)	94	*Gammaproteobacteria*	Fe Red.	Ziegler et al., 2013
562968	*Acidiphilium angustum* (NR_025850.1)	98	*Alphaproteobacteria*	Fe Red.	Hiraishi et al., 1998; Rohwerder and Sand, 2003
4497	*Acidiphilium acidiphilium* (NR_036831.1)	97	*Alphaproteobacteria*	Fe Red./ S Ox.	Hiraishi et al., 1998; Rohwerder and Sand, 2003
227409	*Acidiphilium angustum* (NR_025850.1)	96	*Alphaproteobacteria*	Fe Red.	Hiraishi et al., 1998; Rohwerder and Sand, 2003
NROTU462	*Acidiphilium angustum* (NR_025850.1)	96	*Alphaproteobacteria*	Fe Red.	Hiraishi et al., 1998; Rohwerder and Sand, 2003
219194	*Acidiphilium multivorum* (NR_074327.1)	100	*Alphaproteobacteria*	Fe Red./As Ox.	Wakao et al., 1994; Hiraishi et al., 1998; Rohwerder and Sand, 2003


Of these OTUs, 69 and 92% were more abundant at low pH compared to high pH in the bulk and rhizosphere, respectively. Some exceptions to this trend were all four S-oxidizers and four Fe-reducers (*Acidibacter* [228030 and 4301944], *Metallibacterium* [81089], and *Acidiphilium* [219194]) in the bulk, and two S-oxidizers (*Thioprofundum* [589587 and NROTU388]) in the rhizosphere. A large proportion of the Fe-cyclers/S-oxidizers exhibited a significant negative correlation between their relative abundance and pH in the rhizosphere and bulk, however, more OTUs were negatively correlated with pH in the rhizosphere than in the bulk (25 vs. 14, respectively) (Supplementary Figure 2). Interesting exceptions included OTUs that showed a positive association with pH in the bulk and a negative association in the rhizosphere (the S-oxidizing *Thiomonas* and *Dyella*, and Fe-reducing *Metallibacterium* [81089]) and OTUs that showed a positive association with pH in both bulk and rhizosphere (S-oxidizing *Thioprofundum*). Additionally, there were some OTUs that appeared to have an optimal pH range of 3–6 in the bulk (*Metallibacterium* [540036] and some *Acidibacter* [228030, 4301944, and 1110303]), yet were present and active in the rhizosphere of plants growing in substrate with a pH in the 2–3 range (Plot 19b.R and Plot 5a.R) (Figure 4). One explanation for this is that the buffalo grass plants create a buffering effect in the rhizosphere, a phenomenon which was previously documented (Solís-Dominguez et al., 2011), thus forming more neutral microniches adjacent to the plant roots. Similarly, several acidophilic metal-cyclers that were present in the rhizosphere of plants growing in acidic conditions were also present in very low abundance in the rhizosphere at even the highest pH (7.76) (i.e., *Alicyclobacillus, Leptospirillum, Acidibacter* [NROTU1218], *Aciditerrimonas* [160500], *Acidibacterium* [1003326], *Metallibacterium* [81089], and *Acidiphilium* [227409 and 219194]), signifying that in neutral zones, acidic microniches might also be present, and may actually foreshadow future re-acidification of the area.

Other than the activity of *Acidiphilium* (227409), which was negatively correlated with pH (Supplementary Table 4) in the rhizosphere, there were no other significant correlations after FDR correction. This signifies that some other factor might be associated with bacterial activity, such as TOC which is significantly correlated positively with *Leptospirillum* and *Acidobacterium* and negatively with *Aciditerrimonas* (Supplementary Figure 3).

### N_2_-Fixing and General PGPB

#### Abundance vs. Activity

In bulk materials, N_2_-fixing bacteria and PGPB were present at high pH and nearly absent at low pH. However, in the rhizosphere, some OTUs were present under both the high and low pH conditions (for example, *Devosia* [1053775]) (Figure 4). This pattern indicated that these high abundance OTUs were neutrophilic, and potentially colonizing circum-neutral micro-niches created by the buffering effect produced by buffalo grass roots (Solís-Dominguez et al., 2011). Overall, putative N_2_-fixing bacteria and PGPB were more abundant at high pH. This is supported by a significant positive correlation between all of the OTUs in this group and pH for both rhizosphere and bulk, with the exception of *Arthrobacter* (1081815) (Supplementary Figure 4).

All N_2_-fixing and PGPB OTUs had similar levels of activity in the bulk and rhizosphere (Figure 4). Additionally, the activities did not show any significant correlation with pH after FDR correction (Supplementary Figure 5). Again, another factor could have been more influential on bacterial activity, such as EC which was significantly correlated with two OTUs in the rhizosphere (*Psuedomonas* [829851] and *Hyphomicrobium* [1056070]) (Supplementary Figure 5).

Comparing the presence of PGPB/N_2_-fixers with Fe-cyclers/S-oxidizers at high versus low pH also revealed a distinct pattern. At high pH, the rhizosphere had more PGPB/N_2_-fixers than Fe-cyclers/S-oxidizers while at low pH, both groups were present. In the bulk at high pH there were more PGPB/N_2_-fixers than Fe-cyclers/S-oxidizers, however, at low pH, there were very few PGPB/N_2_-fixers and a dominance of Fe/S-oxidizers and Fe-reducers.

#### nifH Gene

**FIGURE 5:**
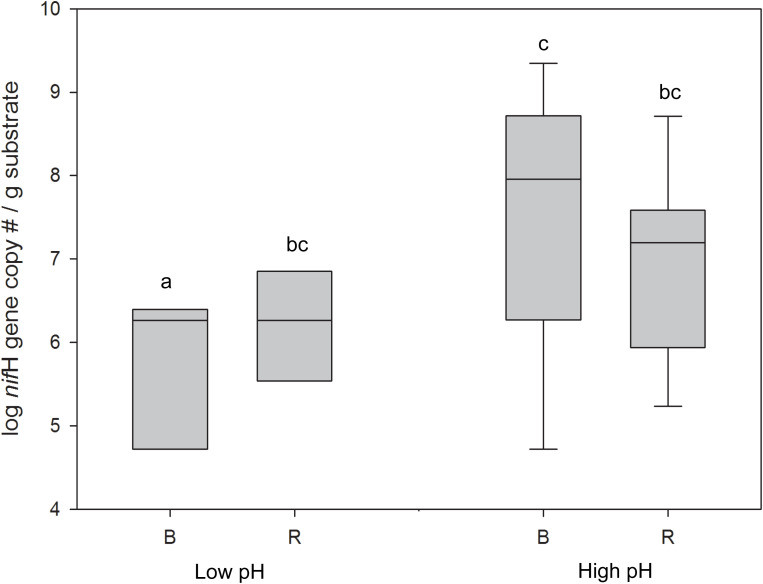
*nif*H gene abundance in buffalo grass bulk and rhizosphere samples from high pH (>4) and low pH (<4) substrate. Different letters indicate significant difference (ANOVA, *p* < 0.05). B, bulk; R, rhizosphere.

qPCR was performed to quantify the *nif*H gene as a measure of the N_2_-fixing potential of the microbial communities from the rhizosphere and bulk samples across a pH gradient. *nif*H gene abundance was significantly greater in the high pH vs. the low pH bulk compartments (ANOVA; *p* < 0.05), however, no significant difference was observed between the high and low pH rhizosphere compartments (Figure 5). At low pH, a significant difference in *nif*H copy number was observed between the rhizosphere and bulk, but this difference was not observed for the high pH substrates. Similarly, there was a stronger correlation between the *nif*H gene abundance and pH in the bulk (ρ = 0.64, *p* < 0.01) than in the rhizosphere (ρ = 0.51, *p* < 0.05).

The lower limit of detection was determined based on the average C_q_ of the extraction blanks, which was 34.42. Two samples, Row 2b.B and Row 4a.B were below this detection limit (average C_q_ of 35.92 and 36.06 respectively). To account for extraction error, they were assigned a C_q_ equivalent to the lower detection limit (34.42). This equated to 3.5 × 10^-2^ copy# μL-extract^-1^, or 5.95 × 10^4^ copy# g-soil^-1^.

## Discussion

The ultimate goal of phytostabilization is the establishment of a plant cover to mitigate contaminant dispersal, however, this can be difficult in metalliferous acid-generating pyritic mine tailings susceptible to re-acidification after initial addition of a neutralizing amendment. Under these conditions, pH as a measure of re-acidification is also indicative of the extent of pyrite weathering and bioavailability of metal(loid)s such as Fe, As, Pb, and Zn (Solís-Dominguez et al., 2011; Valentín-Vargas et al., 2014, 2018). Therefore, determining the microbial community dynamics of the plant rhizosphere and bulk compartments is vital for improved plant establishment because of their critical role in biogeochemical cycling and plant health. In this study, bacterial communities were characterized with a focus on putative PGPB and Fe-cycling/S-oxidizing (acid-generating) bacteria (AGB) from rhizosphere and bulk compartments of buffalo grass during phytostabilization of mine tailings at various stages of re-acidification (pH range: 2.35–7.76). The observed stark shift in rhizosphere and bulk microbial communities with decreasing pH, transitioning from a plant-supporting to an acid-generating community, has strong implications for the ability of the site to sustain long-term plant growth.

**FIGURE 6:**
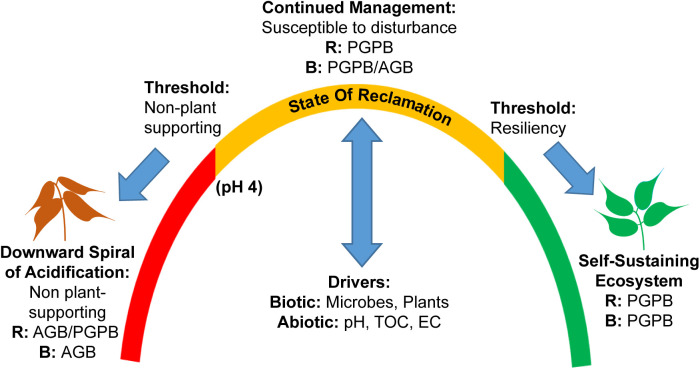
Conceptual model depicting states of reclamation in terms of presence of plant-growth-promoting (i.e., N_2_-fixing) and acid-generating (i.e., Fe/S cycling) bacteria in the bulk and rhizosphere. AGB, acid-generating bacteria; PGPB, plant-growth-promoting bacteria; B, bulk; R, rhizosphere; TOC, total organic carbon.

Bacterial composition and diversity, presence of AGB vs. PGPB, and capacity for N_2_-fixation was strongly linked to pH. Above pH 4, alpha and beta diversity and microbial community composition were similar for the rhizosphere and bulk. Plant-sustaining soils are often characterized by an alpha diversity that is either similar in the rhizosphere and bulk (Edwards et al., 2015) or greater in the bulk than rhizosphere (García-Salamanca et al., 2013). Even in some metal-polluted soils, the alpha diversity of the bulk is greater than the rhizosphere (Navarro-Noya et al., 2010). In addition, beta diversity patterns of typical soils reveal an overlap in the composition of the rhizosphere and bulk microbial communities (Lundberg et al., 2012; Edwards et al., 2015). Thus, the diversity patterns of the rhizosphere and bulk at high pH were similar to that of a typical plant-supporting soil, however the residual presence of AGB in the bulk in combination with the AGP of the tailings, still leaves this region susceptible to re-acidification. Thus, we conclude that these materials have not yet reached a plant-sustaining state (Figure 6).

In contrast, at low pH (<4), a stark difference was observed between the rhizosphere and bulk community composition and diversity patterns. Whereas findings of lower diversity in acidic soils have been reported previously (Lauber et al., 2009; Nacke et al., 2011; Zhalnina et al., 2015; Fan et al., 2018), this is the first report (as far as we know) of lower microbial diversity in the bulk relative to the rhizosphere. Plant root exudates can have a large effect on the rhizosphere microbial community, acting as a selective medium (Berendsen et al., 2012); therefore, we contend that under the highly stressed conditions imposed by low pH, the buffalo grass may be manipulating the rhizosphere community composition relative to the bulk by producing exudates that select for a plant-sustaining community. Another explanation is that the buffering effect of buffalo grass on the rhizosphere (Solís-Dominguez et al., 2011) prevents or delays acidification in this compartment, creating more neutral micro-niches that not only support a higher species diversity, but also may protect some species from the more acidic conditions of the surrounding substrate. Importantly, if the bulk is the source of microbes for the rhizosphere, and the rhizosphere shows a significant and enhanced diversity relative to the bulk (Smalla et al., 2001; Edwards et al., 2015), then it follows that a bulk soil depleted of species diversity provides a compromised pool of microorganisms as a resource for plant-rhizosphere recruitment (Yeoh et al., 2017). Eventually this would lead to isolated plant-associated havens of microbial diversity that are susceptible to a downward spiral of acidification. Ultimately, an overall decrease in ecosystem microbial diversity would result as plants die off along with their associated rhizosphere microbial communities.

### Fe-Cyclers/S-Oxidizers (Acid-Generating Bacteria)

Along the pH continuum from 7.8 to 2.4, the bacterial community shifted from a diverse community containing N_2_-fixing bacteria and other PGPB to one dominated by both lithoautotrophic Fe- and S-oxidizers and heterotrophic Fe-reducers, collectively referred to as AGB. This shift in community that was largely associated with a decrease in pH was also observed in previous studies (Valentín-Vargas et al., 2014,Valentín-Vargas et al., 2018; Honeker et al., 2017).

Fe-oxidizers and Fe-reducers contribute to acidification in pyritic mine tailings directly and indirectly, respectively. Fe- (and S)-oxidizers can directly accelerate acidification because they oxidize pyrite under acidic conditions (Evangelou and Zhang, 1995) which produces even more acidity (H^+^), thus leading to further acidification. However, it is interesting that putative Fe-reducers by far dominated the top 100 OTUs in the rhizosphere and bulk rather than Fe/S oxidizers. Ferric iron respiration is widespread among heterotrophic acidophiles because ferric iron (Fe^3+^) is more bio-available at pH < 3.5 and the redox potential is greater, making it a thermodynamically favored electron accepter (Coupland and Johnson, 2007; Nitschke and Bonnefoy, 2016). In anoxic conditions, such as areas subjected to extended waterlogging or flooding, Fe(III)-reducers have been found to contribute to pH neutralization (Burton et al., 2008). At IKMHSS, however, the semi-arid environment with intermittent rainfall and irrigation events contributes to a heterogeneous profile in the top 25 cm including both oxic and anoxic micro-regions in which microbial Fe-reduction can lead to acidification indirectly via two pathways: (1) by consumption of organic compounds which are toxic to lithoautotrophic Fe-oxidizers (Schippers et al., 2000; Baker and Banfield, 2003; Liu et al., 2011; Méndez-Garcia et al., 2015) thereby facilitating Fe-oxidation; and (2) by rapid cycling of Fe in combination with Fe-oxidizers such as *Acidithiobacillus* *ferrooxidans* (present at low abundance) and *L. ferriphilum* (present at high abundance, see Figure 4) (Küsel et al., 2003). For example, *Acidiphilium* spp., present at low pH in both the rhizosphere and bulk substrates, are known acidophilic iron-respiring bacteria capable of reducing both soluble and precipitated ferric iron species (Küsel et al., 2003; Bilgin et al., 2004).

Whereas *Acidiphilium* spp. were generally more abundant in the bulk substrate, their presence in the rhizosphere at low pH indicates they were potential root-colonizers. Interestingly, Honeker et al. (2017) found that at low pH, there was an increase in relative abundance of *Alphaproteobacteria* on the buffalo grass root surface, which was hypothesized to be *Acidiphilium*. Previous research has shown that *Acidiphilium* spp. are capable of reductive dissolution of ferric Fe minerals, such as ferrihydrite (Bridge and Johnson, 2000), which are known to form plaques on the buffalo grass root surfaces (Honeker et al., 2016). This is consistent with the findings in this study where the relative abundance of all *Acidiphilium* OTUs are higher at low pH (Figure 3) and are significantly negatively correlated with pH (Supplementary Figure 2).

Two other Fe-reducers to note were *Acidobacter ferrireducens* and *Aciditerrimonas ferrireducens*. One *Acdb. ferrireducens* OTU (NROTU1218) was one of the most abundant and active OTUs in the bulk and rhizosphere at low pH (Figure 4). In addition to being As-tolerant, *Acdb. ferrireducens* reduces Fe in the mineral phase, particularly Schwertmannite (Falagán and Johnson, 2014) which is found as a secondary phase mineral following oxidative dissolution of pyrite at IKMHSS (Hayes et al., 2014). Additionally, it is plausible that this gammaproteobacterium may constitute some of the *Gammaproteobacteria* previously shown to colonize root surfaces of buffalo grass growing in more acidic conditions (Honeker et al., 2017). The five of the most active OTUs in the rhizosphere and bulk at low pH were closely related to the genus *Aciditerrimonas* which has only one described species, the Fe-reducer *Acdt. ferrireducens*. There is a lack of discussion of this species in the literature, therefore, further studies are important to determine what role it plays during phytostabilization of pyritic mine tailings.

### N_2_-Fixing and Other PGPB

The long-term success of phytostabilization under such harsh conditions, requires the stable establishment of a plant-growth supporting microbial community containing N_2_-fixing bacteria and other PGPB. All of the most abundant OTUs that are putative N_2_-fixing and general PGPB were present in the high pH rhizosphere and bulk samples signifying the strong presence of a plant-growth supporting community. Most of these OTUs were also present in the rhizosphere of the low pH samples, however, they were conspicuously absent in the low pH bulk samples (Figure 4). This pattern is supported by a significant positive correlation between their relative abundances and pH in the bulk and rhizosphere, with the exception of one OTU closely related to *Arthrobacter pascens* (Supplementary Figure 4) which is discussed in more detail below. The data suggest that as pH decreases, some PGPB can survive in the circum-neutral rhizosphere micro-niches created by the buffalo grass roots (Solís-Dominguez et al., 2011), however, the depletion of PGPB from the bulk at low pH indicates that future plants may struggle due to the lack of available PGPB to recruit from the bulk.

A large proportion of the N_2_-fixing/PGPB OTUs in the rhizosphere and bulk closely matched species within the subphylum *Alphaproteobacteria*, particularly within the order *Rhizobiale*s. OTUs closely matched to *Hyphomicrobium aestuarii* and *Phyllobacterium* *loti* were enriched in the rhizosphere, particularly at low pH, showing that these genera were highly plant-associated. Honeker et al. (2017) found a positive association between the relative abundance of *Alphaproteobacteria* colonizing the root surface of buffalo grass and pH in Phase 3, which was hypothesized to include N_2_-fixing bacteria/PGPB. The results from this study support this hypothesis.

The *Arthrobacter pascens* (OTU1081815) detected in this study is a PGPB species previously found to be beneficial to plants during phytostabilization of mine tailings. *Arthrobacter* inoculation of buffalo grass and quailbush seeds significantly improved plant biomass and increased plant survival in compost-amended mine tailings during a greenhouse study (Grandlic et al., 2008, 2009). Additionally, *Arthrobacter* was significantly associated with quailbush during phytostabilization of mine tailings in a mesocosm study compared to tailings only and tailings with compost treatments (Valentín-Vargas et al., 2018). This species not only has the ability to fix nitrogen, but it also has other PGPB traits such as siderophore and indole acetic acid (IAA) production (Langella et al., 2014) and has been shown to significantly increase plant height, shoot dry weight, and root dry weight (Yu et al., 2012). The presence of acidophilic (De Boer and Kowalchuk, 2001) and metal-tolerant (Margesin and Schinner, 1996; Grandlic et al., 2008) species within this genus also make it an ideal PGPB in the acidic conditions of pyritic mine tailings, as indicated by the fact that it was the one PGPB whose relative abundance was not positively correlated with pH.

The *nif*H gene was quantified to assess the N_2_-fixing capacity of the substrate. *nif*H gene abundance showed a similar pattern to the abundance of N_2_-fixing bacteria and PGPB in the bulk vs. the rhizosphere at high and low pH indicating that (1) the rhizosphere at low pH can sustain neutrophilic N_2_-fixing bacteria, possibly due to a plant-induced buffering effect, and (2) the capacity for N_2_-fixation in the bulk decreases with re-acidification. The decrease in N_2_-fixing capacity of the bulk substrate after re-acidification further confirms the proposed decrease in the plant-growth promoting capabilities of low pH bulk microbial communities.

### Implications for Phytostabilization Management

We present a conceptual model that represents the dynamics of a phytostabilization site and provides useful guidelines for management strategies (Figure 6). In the continuum between an acidic mine tailing environment incapable of supporting plant growth, and a resilient state capable of sustained plant growth, we postulate that there are two thresholds. The first critical threshold occurs at ca. pH 4, below which PGPB diversity and functional capacity is compromised as demonstrated by this study. The second threshold is defined by the point at which biotic (plants, microbes) and abiotic (AGP, pH, TOC, etc.) conditions reach a state of resiliency characterized by a microbial signature of PGPB in both the rhizosphere and bulk, and a lack of AGB in both compartments. Beyond this point, a sustainable ecosystem exists that can resist disturbance. The 15% composted treatment at IKMHSS site reached an intermediary state of reclamation between these two thresholds in which plant growth was supported in the presence of PGPB, but the system was still susceptible to disturbance. The system retained substantial AGB in the bulk and retained a minimal amount in the rhizosphere. A 20% composted treatment at IKMHSS maintained a neutral pH and plant establishment over the duration of the study (41 months) (Gil-Loaiza et al., 2016), possibly representing conditions of greater resiliency. This treatment is the subject of future microbial analysis.

Choice of management strategies should be geared toward an ultimate goal of ecological stability. However, for sites with extreme conditions similar to IKMHSS, a continued management approach would be a likely requirement in order to sustain plant growth. A proposed critical management strategy is to ensure that the pH remains above 4, to avoid the downward spiral characterized by the transition to an AGB dominated community with declining abundance of PGPB. This can be done by (1) re-applying compost, (2) adding lime, and (3) preventing erosion of critical amendments. In conclusion, continued monitoring of biogeochemical conditions (microbial communities, pH, EC, TOC, etc.) is necessary until stability is reached.

## Conclusion

A drastic change in microbial diversity and community composition of buffalo grass rhizosphere and bulk substrate was observed across a pH gradient during phytostabilization of metalliferous pyritic mine tailings. Rhizosphere and bulk substrates above pH 4, were characterized by microbial communities with a higher diversity and abundance of PGPB and neutrophilic N_2_-fixers. As the pH decreased below 4, community diversity decreased and acidification of the substrate was driven by increasingly more abundant lithoautotrophic Fe/S-oxidizers and heterotrophic Fe-reducers whose activity was detrimental to long-term phytostabilization success. The PGPB and N-fixers observed above pH 4 were virtually absent from the bulk under low pH conditions, but were still present in low abundance in the rhizosphere, presumably due to the buffering effect produced by buffalo grass roots. These PGPB may help plant survival under the extreme acidic conditions. However, as plants die along with their associated havens of microbial diversity, the tailings lose the capacity to support plant growth due to the virtual absence of PGPB and N_2_-fixing populations in the low pH bulk substrate.

## Author Contributions

LH conceived this study, performed microbiota analysis, statistically analyzed all data, and wrote the manuscript. CG performed qPCR. JN and RM contributed to data interpretation and manuscript writing. JC and RM conceived the larger IKMHSS study. All authors reviewed the manuscript.

## Conflict of Interest Statement

The authors declare that the research was conducted in the absence of any commercial or financial relationships that could be construed as a potential conflict of interest.

## Footnotes

1
www.qiime.org

2
https://blast.ncbi.nlm.nih.gov/Blast.cgi

3
http://huttenhower.sph.harvard.edu/galaxy
