# Supplementary material for: Effect of Re-acidification on Buffalo Grass Rhizosphere and Bulk Microbial Communities During Phytostabilization of Metalliferous Mine Tailings
Source: Front Microbiol. 2019 May 31;10:1209. doi: 10.3389/fmicb.2019.01209 (PMC6554433; doi:10.3389/fmicb.2019.01209)
Supplement: Supplementary file 1 [file Presentation_1.PPTX]

## Slide 1
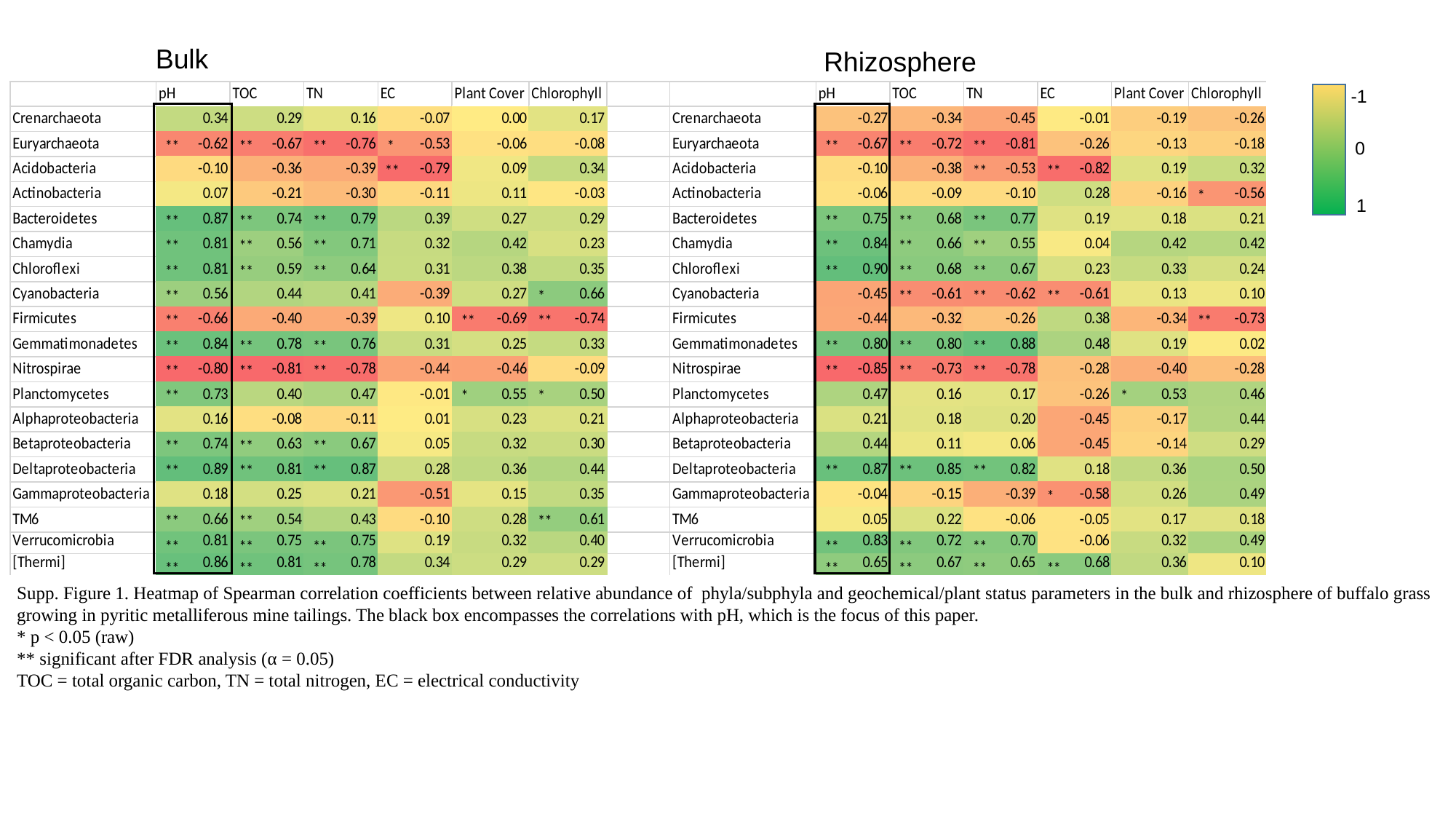

Bulk
Rhizosphere
 -1
0
 1
Supp. Figure 1. Heatmap of Spearman correlation coefficients between relative abundance of phyla/subphyla and geochemical/plant status parameters in the bulk and rhizosphere of buffalo grass growing in pyritic metalliferous mine tailings. The black box encompasses the correlations with pH, which is the focus of this paper.
* p < 0.05 (raw)
** significant after FDR analysis (α = 0.05)
TOC = total organic carbon, TN = total nitrogen, EC = electrical conductivity

## Slide 2
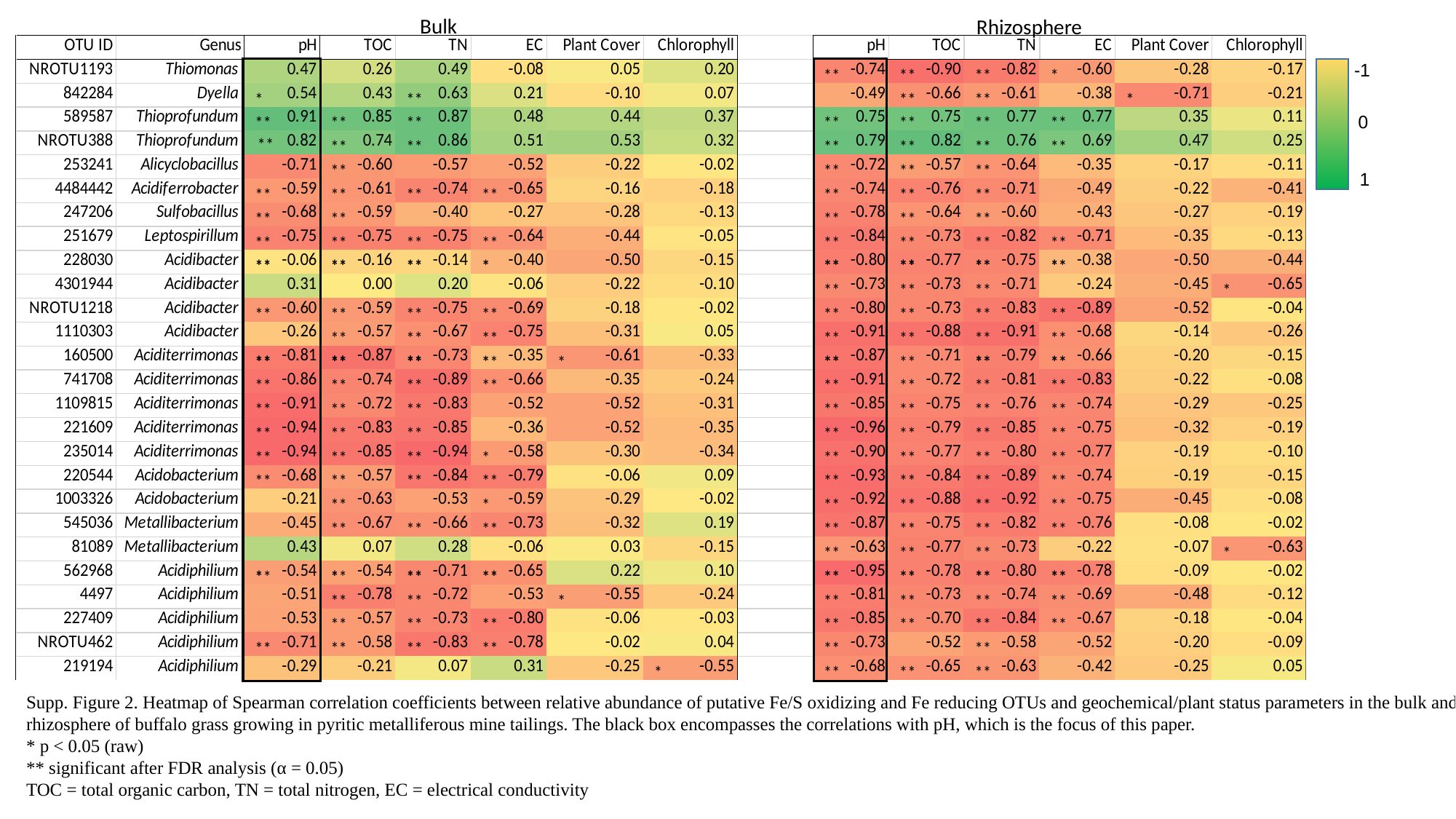

Bulk
Rhizosphere
 -1
0
 1
Supp. Figure 2. Heatmap of Spearman correlation coefficients between relative abundance of putative Fe/S oxidizing and Fe reducing OTUs and geochemical/plant status parameters in the bulk and rhizosphere of buffalo grass growing in pyritic metalliferous mine tailings. The black box encompasses the correlations with pH, which is the focus of this paper.
* p < 0.05 (raw)
** significant after FDR analysis (α = 0.05)
TOC = total organic carbon, TN = total nitrogen, EC = electrical conductivity

## Slide 3
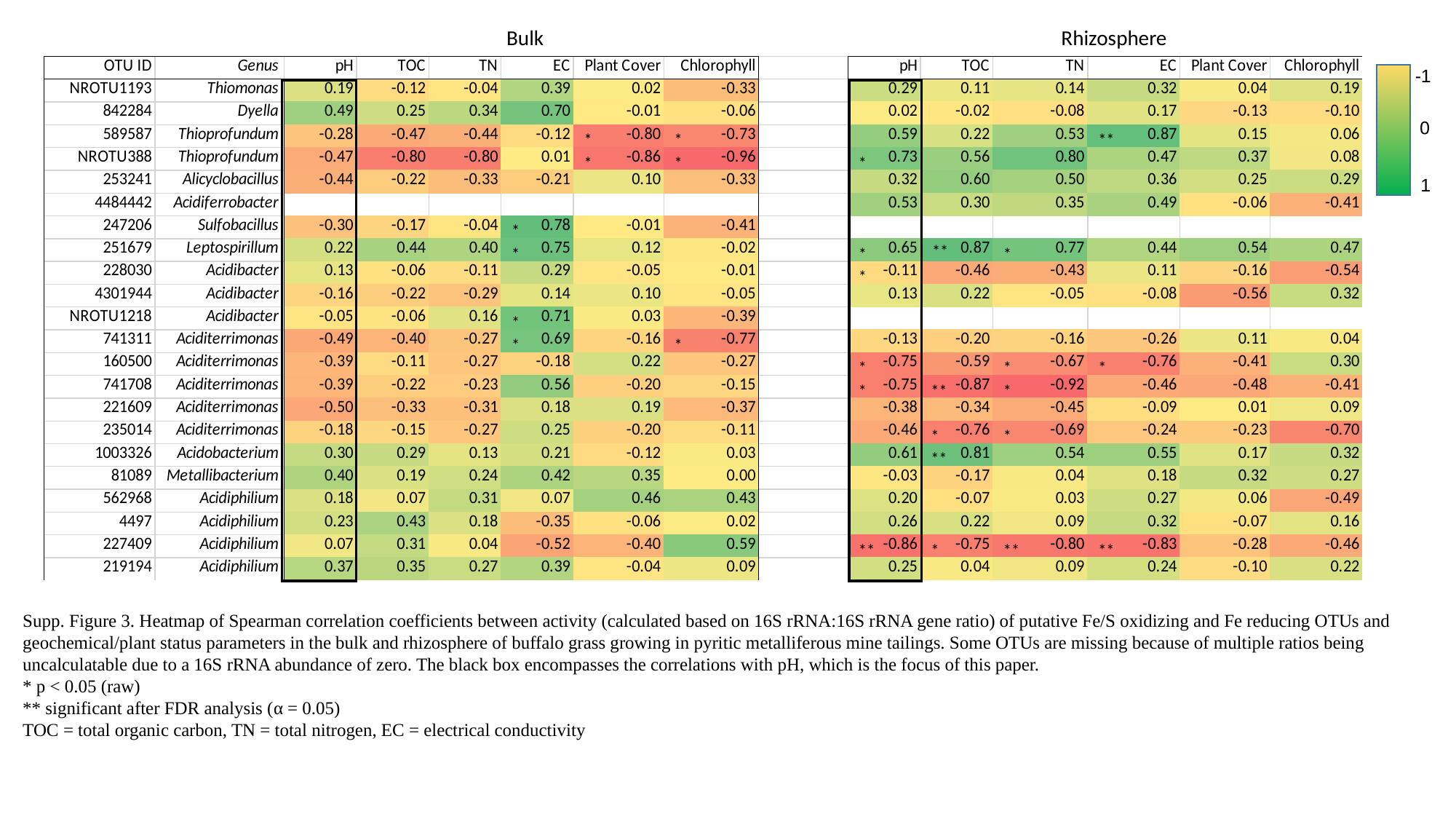

Rhizosphere
Bulk
 -1
0
 1
Supp. Figure 3. Heatmap of Spearman correlation coefficients between activity (calculated based on 16S rRNA:16S rRNA gene ratio) of putative Fe/S oxidizing and Fe reducing OTUs and geochemical/plant status parameters in the bulk and rhizosphere of buffalo grass growing in pyritic metalliferous mine tailings. Some OTUs are missing because of multiple ratios being uncalculatable due to a 16S rRNA abundance of zero. The black box encompasses the correlations with pH, which is the focus of this paper.
* p < 0.05 (raw)
** significant after FDR analysis (α = 0.05)
TOC = total organic carbon, TN = total nitrogen, EC = electrical conductivity

## Slide 4
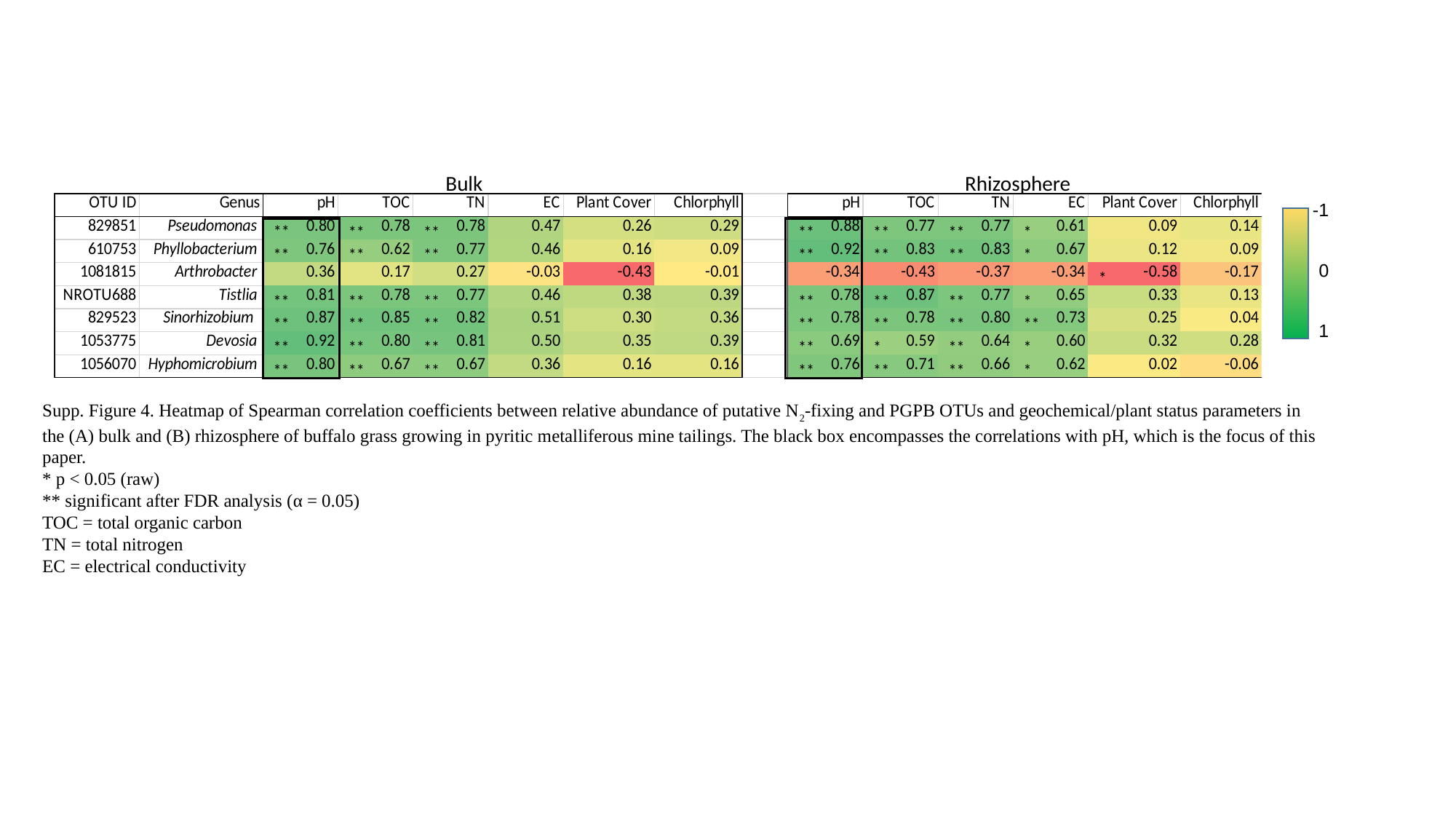

Bulk
Rhizosphere
-1
0
1
Supp. Figure 4. Heatmap of Spearman correlation coefficients between relative abundance of putative N2-fixing and PGPB OTUs and geochemical/plant status parameters in the (A) bulk and (B) rhizosphere of buffalo grass growing in pyritic metalliferous mine tailings. The black box encompasses the correlations with pH, which is the focus of this paper.
* p < 0.05 (raw)
** significant after FDR analysis (α = 0.05)
TOC = total organic carbon
TN = total nitrogen
EC = electrical conductivity

## Slide 5
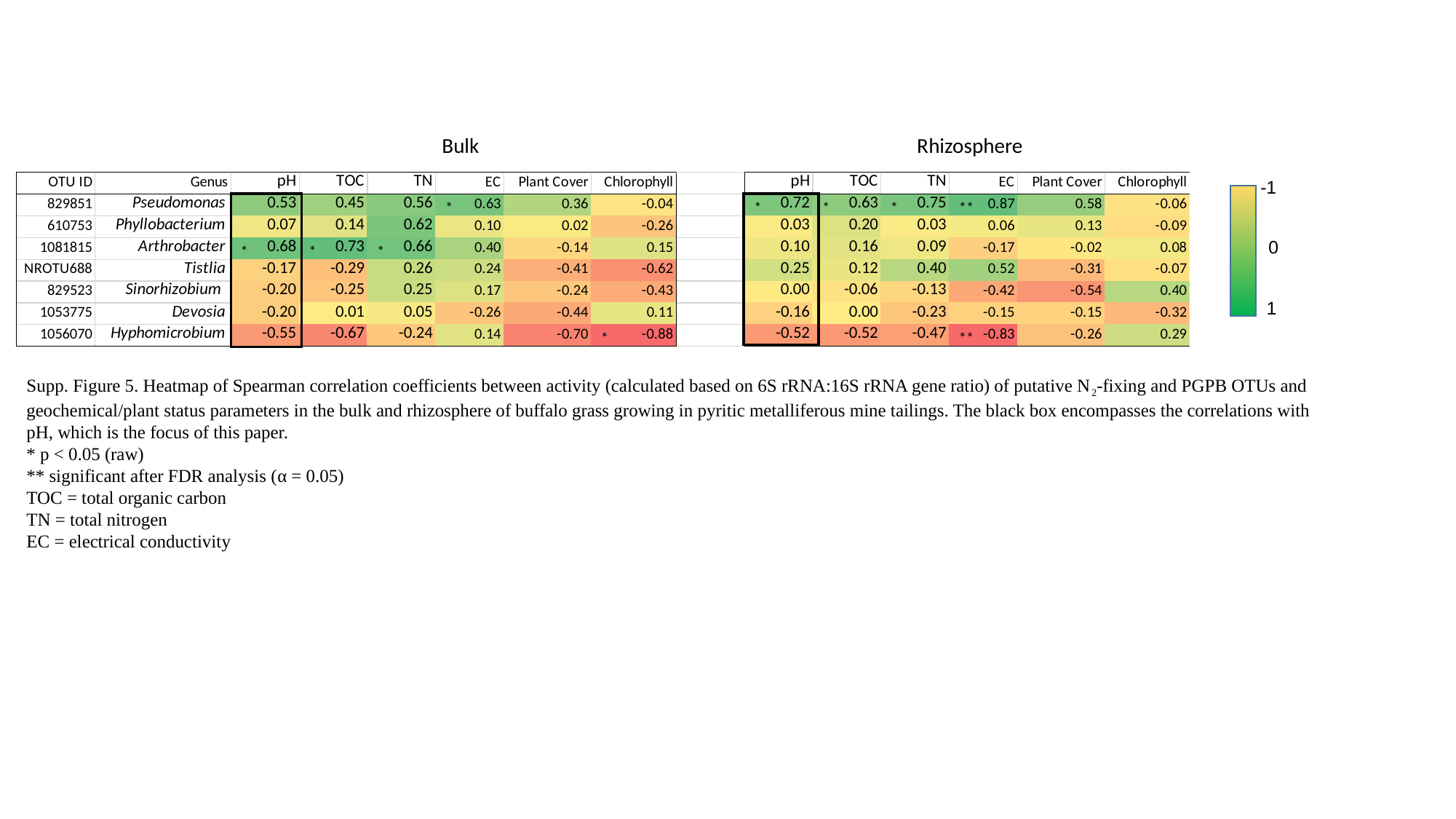

Bulk
Rhizosphere
-1
0
1
Supp. Figure 5. Heatmap of Spearman correlation coefficients between activity (calculated based on 6S rRNA:16S rRNA gene ratio) of putative N2-fixing and PGPB OTUs and geochemical/plant status parameters in the bulk and rhizosphere of buffalo grass growing in pyritic metalliferous mine tailings. The black box encompasses the correlations with pH, which is the focus of this paper.
* p < 0.05 (raw)
** significant after FDR analysis (α = 0.05)
TOC = total organic carbon
TN = total nitrogen
EC = electrical conductivity
